# Hokusai: beyond the Great Wave

**DOI:** 10.1080/17571472.2018.1486504

**Published:** 2018-06-13

**Authors:** Francesco Carelli

**Affiliations:** a Family Medicine, University of Milan, Milan, Italy

Katsushika Hokusai (1760–1849) has long enjoyed a strong international reputation and is considered by many to be Japan’s greatest artist.

The British Museum staged in 2017 the first exhibition in the UK to focus on his later years’ life and art. This features his iconic print ‘The Great Wave’ [c. 1831], and continuing to the sublime painted works produced right up to his death at the age of 90. Supported by the Mitsubishi Corporation, *Hokusai: beyond the Great Wave* has provided new insight into the prodigiously productive last thirty years of Hokusai’s life and art from around 1820–1849.

The exhibition has adopted a new approach to explore Hokusai’s later career in thematic as well as chronological terms. The exhibition has shed light on Hokusai’s personal beliefs, as well as his spiritual and artistic quest through major paintings, drawings, woodblock prints and illustrated books. Many have never been seen before in the UK and indeed can only be displayed for a limited length of time. Half the artworks have been rotated midway through the exhibition run for conservation reasons. Due to their light sensitivity some works can only be displayed for a limited amount of time, and this preserves their vivid colours. Each rotation tells the same story, but with the opportunity to see a selection of different works. The exhibition featured around 110 works in each rotation. From iconic landscapes and wave pictures to deities and mythological beasts, from flora and fauna to beautiful women, from collaborations with other painters and writers to still lives, the works on show are extraordinarily varied, with objects drawn from the British Museum’s superb collection and many loans from Japan, Europe and the United States.

*The Great Wave* was acquired in 2008 by the British Museum with the assistance of the Art Fund. Hokusai created this world renowned masterpiece when he was about seventy. Mt Fuji and its wider spiritual significance was a model for Hokusai in his quest for immortality during his later years.

The print series *Thirty*-*Six Views of Mt Fuji* (published around 1831–33) revived Hokusai’s career after some personal challenges of the late 1820s. *The Great Wave*, with its use of deep perspective and imported Prussian blue pigment, reflects how Hokusai adapted and experimented with European artistic style.

Also shown was a rare group of paintings from the National Museum of Ethnology, Leiden, done in a unique European influenced style, which were commissioned from Hokusai by employees of the Dutch East India Company in about 1824–1826. Throughout his career, and particularly in the later years, Hokusai’s paintings brought vividly to life an extraordinary bestiary of dragons, Chinese lions, phoenixes and eagles, and forcefully energised depictions of mythological figures and holy men. He published numerous brush drawing manuals, notably Hokusai *manga* (Hokusai’s Sketches, 15 vols, 1814–1878) which spread his artistic style and reputation widely.

Hokusai based his exploration of the outside world on his subjective identification with his surroundings rather than any objectively ‘scientific’ or technical approach. For Hokusai and his contemporaries, the perceived world could connect seamlessly with a world of powerful ‘unseen’ forces and agencies. Ghosts and vengeful spirits inhabited a closely parallel world that was believed could easily spill into ours. The artist considered that he was passing on ‘divine teachings’ to his pupils, to craft artists and to the world.

The exhibition has also displayed a magnificent hanging loan from the Metropolitan Museum in New York: *Red Shōki, the demon*-*queller*, who could protect your home against the scourge of smallpox.

In the late 1820s Hokusai suffered many personal challenges, including the death of his wife, illness, and financial woes caused by an errant grandson. His daughter Eijo [art name Ōi, c1800–1857], herself an accomplished artist, quit an unsuccessful marriage to return and care for her aged father, and to work with and alongside him. The exhibition revealed their modest living circumstances, displaying their portraits and drawing on the recollections of Hokusai’s pupils.

From his eighty-eighth year until his death, Hokusai’s extraordinary last painted works show that the artist had indeed reached a sublime realm in his beliefs and art. He fervently believed that his skills as an artist would continue to improve the older he got. We can only but agree.

## Permissions

I was given verbal permission to use parts of the British Museum brochure of this exhibition to prepare this paper.

## Disclosure statement

No potential conflict of interest was reported by the author.

